# Multi-Agent Deep Reinforcement Learning Based Dynamic Task Offloading in a Device-to-Device Mobile-Edge Computing Network to Minimize Average Task Delay with Deadline Constraints

**DOI:** 10.3390/s24165141

**Published:** 2024-08-08

**Authors:** Huaiwen He, Xiangdong Yang, Xin Mi, Hong Shen, Xuefeng Liao

**Affiliations:** 1 School of Computer, Zhongshan Institute, University of Electronic Science and Technology of China, Zhongshan 528400, China; he_huai_wen@aliyun.com (H.H.); yangxiangdong.cs@aliyun.com (X.Y.); 202021080226@std.uest.edu.cn (X.M.); 2 Computer Science and Engineering School, University of Electronic Science and Technology of China, Chengdu 611731, China; 3 Engineering and Technology, Central Queensland University, Rockhampton 4701, Australia; h.shen@cqu.edu.au; 4 School of Data Science and Artificial Intelligence, Wenzhou University of Technology, Wenzhou 325027, China

**Keywords:** mobile edge computing, dynamic matching, D2D, delay constraint, multi-agent reinforcement learning

## Abstract

Device-to-device (D2D) is a pivotal technology in the next generation of communication, allowing for direct task offloading between mobile devices (MDs) to improve the efficient utilization of idle resources. This paper proposes a novel algorithm for dynamic task offloading between the active MDs and the idle MDs in a D2D–MEC (mobile edge computing) system by deploying multi-agent deep reinforcement learning (DRL) to minimize the long-term average delay of delay-sensitive tasks under deadline constraints. Our core innovation is a dynamic partitioning scheme for idle and active devices in the D2D–MEC system, accounting for stochastic task arrivals and multi-time-slot task execution, which has been insufficiently explored in the existing literature. We adopt a queue-based system to formulate a dynamic task offloading optimization problem. To address the challenges of large action space and the coupling of actions across time slots, we model the problem as a Markov decision process (MDP) and perform multi-agent DRL through multi-agent proximal policy optimization (MAPPO). We employ a centralized training with decentralized execution (CTDE) framework to enable each MD to make offloading decisions solely based on its local system state. Extensive simulations demonstrate the efficiency and fast convergence of our algorithm. In comparison to the existing sub-optimal results deploying single-agent DRL, our algorithm reduces the average task completion delay by 11.0% and the ratio of dropped tasks by 17.0%. Our proposed algorithm is particularly pertinent to sensor networks, where mobile devices equipped with sensors generate a substantial volume of data that requires timely processing to ensure quality of experience (QoE) and meet the service-level agreements (SLAs) of delay-sensitive applications.

## 1. Introduction

With the advancement of Internet of Things (IoT) technology, sensor networks play an increasingly important role in various applications. The vast amount of data generated by these sensor networks requires real-time processing to support applications such as smart homes, smart cities, and Industrial Internet of Things (IIoT). However, transmitting all data to remote cloud servers for processing presents challenges of high latency and bandwidth limitations. Mobile edge computing (MEC) has emerged as a promising computing paradigm that aims to reduce response time for computation tasks and enhance the quality of experience (QoE) of users by offloading tasks to edge servers [1]. However, the dynamic and random nature of task arrivals can lead to a significant increase in workload during certain periods. This surge in workload poses challenges for edge servers, making it difficult to meet the latency requirements of tasks like face recognition, virtual reality, augmented reality, online games, and more. D2D technology addresses this issue by enabling MDs to offload tasks directly to idle MDs, facilitating resource utilization and collaboration across the network [2].

In D2D–MEC networks, MDs typically operate in two modes: requester MDs (active devices) and server MDs (idle devices) [2]. Requester MDs can offload tasks to server MDs or edge servers, while server MDs not only handle their own tasks but also accept tasks from other requester MDs. This approach can effectively utilize idle resources within the network by enabling task offloading and collaboration among mobile devices. However, most existing research in this field primarily emphasizes the static partitioning of MDs, where active devices and idle devices are pre-determined [3]. This restricts the flexibility of D2D communication in real-world scenarios. Additionally, the dynamic nature of task arrivals and the need for collaboration among active devices present considerable challenges for task offloading in D2D–MEC.

In order to ensure a high quality of experience (QoE) for users in mobile edge computing (MEC) systems, it is crucial to process tasks within their deadlines, especially for delay-sensitive tasks. Zuo et al. [4] proposed an alternating iterative algorithm, based on continuous relaxation and greedy rounding (CRGR), to achieve the Nash equilibrium. Abbas et al. [5] introduced an algorithm that maximizes the number of completed tasks through hierarchical and iterative allocation while minimizing energy consumption and monetary costs. However, the aforementioned studies did not consider leveraging D2D technology to improve the utilization of network resources. Hamdi et al. [6] put forth a layered optimization method to maximize energy efficiency (EE) in D2D–MEC systems under delay constraints. Sun et al. [7] proposed an algorithm to maximize the network energy efficiency in D2D–MEC networks. However, they consider the division of D2D roles to be static.

DRL algorithms have been successfully applied to the task offloading problem in MEC in various existing works [8,9,10]. Huang et al. [8] proposed learning algorithms based on Q-learning or deep Q-learning network (DQN) for task offloading in MEC systems. Luo et al. [11] introduced a distributed learning algorithm based on the asynchronous advantage actor–critic (A3C) technique to optimize energy consumption and quality of experience in software-defined mobile edge networks. Qiao et al. [12] and Li et al. [10] designed a reinforcement learning framework for D2D edge computing and networks to address challenges related to the dynamic nature and uncertainty of the environment. However, most of the aforementioned studies predominantly utilized single-agent reinforcement learning algorithms or overlooked the unknown load dynamics at each mobile device. Due to the dynamical and random nature of MEC systems, the decision space size increases exponentially with the number of mobile devices, which may bring the curse of dimensionality and a struggle to converge for single-agent DRL algorithms.

In this paper, we investigate the dynamic task offloading problem in a D2D–MEC system for delay-sensitive tasks under delay constraints. Our objective is to minimize the long-term average task delay under deadline constraints. To achieve this, we propose a dynamic D2D partitioning approach for MDs, based on a queuing-based system, considering the dynamic load level of MDs and the presence of multiple time slot tasks. To tackle the challenges posed by a huge decision space and coupling of actions across time slots, we formulate the problem as a cooperative Markov game and propose a multi-agent (MA) DRL-based algorithm based on the MAPPO [13] technique and implementation in a CDTE-based manner. Our main contributions can be summarized as follows:

- We introduce a novel dynamic D2D partitioning method based on a queuing system for handling delay-sensitive tasks in D2D–MEC networks. Furthermore, we formulate the problem of minimizing the long-term average task delay under deadline constraints as a dynamic assignment problem, considering the random load level at MDs and multi-slot spanned tasks. Our proposed model surpasses existing approaches by providing a more precise characterization of task latency and improving the utilization of network computing resources. Additionally, it exhibits superior scalability and practicality.
- We formulate the dynamic offloading problem as a cooperative Markov game and propose a multi-agent DRL-based algorithm utilizing the MAPPO technique to address the exponential growth of the decision space. Our proposed algorithm allows for efficient online task decision-making in dynamic and volatile network environments, leveraging the strengths of MAPPO to manage large decision spaces effectively.
- We adopt the CDTE framework, enabling a distributed decision-making model. During the execution phase, each MD can independently make decisions based on its own system state, significantly reducing communication overhead. This reliance on local observations ensures that the system remains robust and adaptable, even in fluctuating network conditions.
- We conduct comprehensive experiments and the numerical results demonstrate the effectiveness and fast convergence of our proposed algorithm in a time-varying system environment. Compared to the sub-optimal outcomes obtained by deploying single-agent DRL, our algorithm, which enables distributed decision-making, achieves a significant reduction of 11.0% in average task completion delay and a 17.0% decrease in the ratio of dropped tasks.

The rest of this paper is organized as follows. Section 2 presents a review of related works. In Section 3, we provide details of the system model. In Section 4, we formulate the task offloading problem with delay constraints as a dynamic assignment problem. In Section 5, we propose a multi-agent DRL-based algorithm based on the MAPPO technique. The experimental setup and numerical results are presented in Section 6. Finally, Section 7 concludes this paper.

## 2. Related Works

### 2.1. Delay-Sensitive Task Offloading in MEC Networks

Research on optimizing latency reduction for delay-sensitive tasks in MEC networks has attracted a great deal of attention [14,15,16,17,18]. Wu et al. [14] introduced a delay-aware energy-efficient (DAEE) online offloading algorithm designed to optimize task offloading in Industrial Internet of Things (IIoT) systems, addressing the challenge of balancing low latency and low power consumption for delay-sensitive and compute-intensive devices. Zhao et al. [17] proposes a novel deep reinforcement learning algorithm for privacy-preserving task offloading in multi-user MEC systems, balancing low latency with the protection of users’ location and usage pattern privacy. Yang et al. [19] proposed a novel offloading framework for the multi-server MEC network to jointly optimize job offloading decisions and computing resource allocation using multi-task learning. Despite the extensive research, most of these studies assume that tasks can be completed within a single time slot. Tang and Wong [20] proposed an algorithm focused on minimizing long-term costs in MEC, taking into account non-divisible tasks that are sensitive to delay and the varying edge loads over multiple time slots. However, their approach did not incorporate the use of D2D communication and did not take into account MADRL-based algorithms.

### 2.2. D2D Communication Applied in MEC Networks

D2D communication, a cornerstone innovation of 5G technology, enables users to delegate tasks to nearby idle devices, significantly boosting the collective computational performance of the network [21,22,23]. Wang et al. [15] proposes a novel algorithm leveraging a Knapsack problem-based pre-allocation strategy and an alternate optimization technique to optimize the energy consumption and transmission delay in D2D-assisted MEC systems. Chai et al. [24] proposed a heuristic algorithm based on the Kuhn–Munkres algorithm and Lagrangian dual method for joint computation offloading and resource allocation in D2D–MEC systems. Sun et al. [7] leveraged the Lyapunov optimization technique to design the DACORA algorithm, in order to maximize long-term utility energy efficiency (UEE), considering the dynamics of task arrival rate and battery level. However, previous research typically regards the roles of D2D devices as service providers or requesters as static, overlooking the dynamic shifts in device roles within the evolving D2D network context. Peng et al. [1] took into account the dynamic partitioning of devices, and proposed an online resource coordinating and allocating scheme based on the Lyapunov optimization framework. However, they overlooked the scenario of tasks spanning multiple time slots and the associated waiting times in the execution process, which hinders the enhanced user experience tailored to user needs.

### 2.3. DRL for Computation Offloading in MEC Networks

DRL has recently emerged as a promising alternative for solving online computation offloading problems in MEC networks. Due to its ability to extract valuable knowledge from the environment and make adaptive decisions, DRL technology has received significant attention recently for edge computing task offloading [8,20,25,26]. Chen et al. [25] first proposed a DQN-based algorithm to handle huge state spaces and learn the optimal computation offloading policy. Wang et al. [27] proposed a task-offloading algorithm based on meta-reinforcement learning to enable the model to quickly adapt to different environments. However, the above research works were based on single agents and made centralized decisions, which may be impractical in the real world as the number of MDs is increasing due to the exploration of the decision space in MEC systems. Some works based on multi-agent DRL schemes are proposed for task offloading problems [28,29,30]. However, most of these approaches rely on the multi-agent deep deterministic policy gradient (MDDPG) framework [31], which is designed for continuous action spaces. This makes it suitable for scenarios where agents need to perform smooth and continuous actions, such as in robotic control. Therefore, the MDDPG algorithm is not well-suited for the target device selection and matching in dynamic D2D task offloading, as studied in this paper.

### 2.4. Federated Learning in MEC Task Offloading

In recent years, federated learning has gained significant attention in edge computing task offloading by enabling multiple devices to collaboratively train models without sharing local data, offering a privacy-preserving solution. To relieve the straggler effect, Ji et al. [32] proposed an edge-assisted federated learning Computation Offloading scheme, in which the stragglers offload partial computation and the offload size were optimized by a proposed threshold-based offloading strategy. To benefit convergence speed and global-model accuracy, Mills et al. [33] introduced non-federated batch normalization layers into federated deep neural networks and allowed users to train models with their own personalized data. Han et al. [34] proposed a federated learning-based training scheme to ease the training burden on each IoT device. To facilitate applying data and resource heterogeneity to federated learning in edge task offloading, Ma et al. [35] integrated information entropy into edge datasets and considered device heterogeneity. However, federated learning has drawbacks. It requires a high communication overhead due to frequent model updates between devices and the central server, which can be problematic in bandwidth-limited environments. Additionally, it suffers from data heterogeneity, where varying data distributions across devices impact the global model’s performance.

Different from the previous studies, this paper introduces a multi-agent reinforcement learning approach tailored for the scheduling of delay-sensitive tasks within D2D–MEC networks. Our algorithm is designed to minimize the completion time of tasks, accounting for their multi-time slot nature and associated deadlines. Distinguishing our work from [8], who focused solely on single-agent reinforcement learning and neglected the collaborative potential of D2D technology among terminal nodes, we also address the critical aspect of user task processing delays. Contrary to their aim of maximizing data processing rates, our focus extends to enhancing the overall user experience.

We select MAPPO as the algorithmic framework for the following compelling reasons: (1) MAPPO’s design is particularly suited for discrete action spaces, which is essential for our scenario wherein decisions involve selecting from a finite set of options, such as offloading tasks to specific D2D service nodes or edge servers—unlike MDDPG, which is better adapted to continuous action spaces. (2) Introduced more recently than MDDPG, MAPPO offers advanced policy optimization and stability in training, allowing for efficient exploration within a large discrete action space and maintaining a critical balance between exploration and exploitation. (3) Our approach leverages MAPPO’s strengths in a discrete action space and introduces a sophisticated, adaptive mechanism for task offloading that responds to real-time network dynamics, effectively minimizing average task delay under strict deadlines—a significant advancement over existing methods.

## 3. System Model

**Figure 1:**
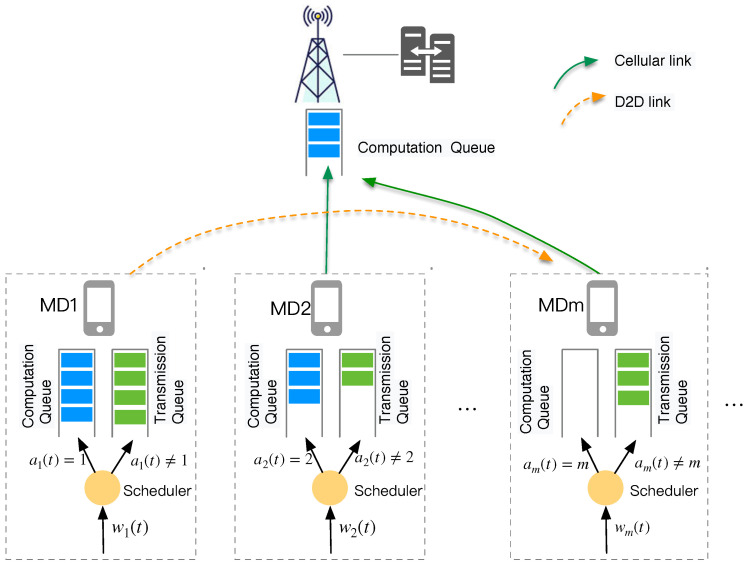
Architecture of D2D–MEC network.

In this paper, we consider a heterogeneous MEC network system consisting of *D* mobile devices and an edge server with D2D communication support, which is denoted as D={0,1,2,…,D}, where 0 represents the edge server. The system architecture is illustrated in Figure 1. We assume that the D2D–MEC system operates in discrete time slots represented by T={1,2,…,T}, where each time slot lasts for Δt seconds. In each time slot, tasks are generated in each MD according to a certain probability model.

In the following subsections, we present the details of the device model, computation model, transmission model, energy model, and delay model employed in the system. The key notation used in this paper is summarized in Table 1.

To accurately mode the service time of the computation task, we adopt a queuing system to represent the task processing procedure. Each MD d∈[1,D] has two types of queues: the computation queue $Q_{d}^{comp}$ used for task execution and the transmission queue $Q_{d}^{tran}$ used to offload tasks to D2D devices or the edge server. For the edge server, there only exists one computation queue, denoted as $Q_{0}^{comp}$.

Since the load of MDs varies stochastically, we dynamically divide the MD set into idle devices (requesters) and active devices (servers) based on the backlog of their computation queues, which are defined as follows:

(1) Idle device: In time slot *t*, $Q_{d}^{comp}\left(t\right)=\emptyset$. Idle devices can provide computing services for other MDs via a D2D link.
(2) Active device: In time slot *t*, $Q_{d}^{comp}\left(t\right)\neq \emptyset$. Active devices only process a task locally, or offload tasks to edge servers or idle MDs, but cannot accept tasks from other MDs.

### 3.1. Task Model

We consider time-sensitive tasks, such as virtual reality (VR) or animation rendering, that can span over multiple time slots. At time *t*, each MD *m* stochastically generates a computation task, denoted as $w_{m}\left(t\right)$, specified by a three-tuple $<s_{m}^{t},c_{m}^{t},\tau_{m}^{t}>$, where $s_{m}^{t}$ represents the size of task *w* in 1-bit unit, $c_{m}^{t}$ denotes the computation complexity of task *w* measured in the number of central processing unit (CPU) cycles required for a 1-bit operation, and $\tau_{m}^{t}$ indicates the task deadline for completion (an integer multiple of the time slot). Each job should be processed within $\tau_{m}^{t}$ time slot to avoid incurring an expiration penalty.

We assume that each task is indivisible, such that the system adopts a binary offloading mode, which allows tasks to be executed either locally or offloaded to an edge server through cellular links, or transferred to idle devices via D2D links. Each MD employs a scheduler that determines the target device for task execution. The active devices set at slot *t* is denoted as $\mathrm{AD}\left(t\right)=\left\{ad_{1},ad_{2},\ldots ,ad_{\left|\mathrm{AD}(t)\right|}\right\}$, and the idle devices at slot *t* is denoted as $\mathrm{ID}\left(t\right)=\left\{id_{0},id_{1},id_{2},\ldots ,id_{\left|\mathrm{ID}(t\right)}|\right\}$, where $id_{0}$ represents the edge server which is always regarded as an idle device due to its sufficient computing resources. Hence, we have |AD(t)|+|ID(t)|=D.

### 3.2. Computation Model

The computation queue follows the first-in-first-out (FIFO) principle, where tasks in the queue buffer must wait for the completion of the preceding task before being scheduled. The computing delay of a task is composed of two parts: the waiting time and execution time.

Let $\tilde{t}$ denote the time slot of task $w_{m}\left(t\right)$ placed in the queue $Q_{d}^{comp}$ at device d∈D, and $l_{m,d}^{comp}\left(t\right)$ denote the time slot of task $w_{m}\left(t\right)$ to be completely processed or dropped in device *d*. Note that, if the task is computed locally, we obtain $\tilde{t}=t$, and $l_{m,d}^{comp}\left(t\right)$ can be written as $l_{m}^{comp}\left(t\right)$. Hence, the number of time slots that task $w_{m}\left(t\right)$ will wait for scheduling before executing it in the computation queue $Q_{d}^{comp}$ can be expressed as follows:

(1) $$\phi_{m,d}^{comp}\left(t\right)={\left[\max_{t^{\prime }\in \left\{0,1,\cdots ,t-1\right\},d^{\prime }\in \left[1,D\right]}l_{d^{\prime },d}^{comp}\left(t^{\prime }\right)-\tilde{t}+1\right]}^{+}$$

where the operator ${\left[x\right]}^{+}=max\left\{0,x\right\}$; $l_{d^{\prime },d}^{comp}\left(0\right)$ is initially set to zero for presentation simplicity. The term $\max_{t^{\prime }\in \left\{0,1,\cdots ,t-1\right\},d^{\prime }\in \left[1,D\right]}l_{d^{\prime },d}^{comp}\left(t^{\prime }\right)$ determines the time slot when all the tasks placed in the computation queue $Q_{d}^{comp}$ before time slot $\tilde{t}$ have either been processing or dropped. Hence, $\phi_{m,d}^{comp}\left(t\right)$ determines the number of waiting time slots in the computation queue.

For each task $w_{m}\left(t\right)$ that arrives at the computation queue $Q_{d}^{comp}$ at the beginning of time slot $\tilde{t}$, it will either be processed completely or dropped in slot $l_{m,d}^{comp}\left(t\right)$, as follows:

(2) $$\begin{array}{c} l_{m,d}^{comp}\left(t\right)=\min\{\tilde{t}+\phi_{m,d}^{comp}\left(t\right)+\left\lceil \frac{s_{m}^{t}c_{m}^{t}}{f_{d}\Delta t}\right\rceil -1,t+\tau_{m}^{t}-1\} \end{array}$$

where $f_{d}$ represents the process rate of device *d*. Specifically, task $w_{m}\left(t\right)$ will be scheduled to be executed at the beginning of slot $\tilde{t}+\phi_{m,d}^{comp}\left(t\right)$. $\left\lceil \cdot \right\rceil$ is the ceiling function, which means devices only switch to executing the next task at the beginning of a new time slot. The term $\left\lceil \frac{s_{m}^{t}c_{m}^{t}}{f_{d}\Delta t}\right\rceil$ represents the total time slots required to process the task completely. Considering the deadline of the tasks, here, we use a min operator to determine the minor value of the process completely and the drop time slot.

When multiple offloaded tasks or a newly generated task arrive at device *d* in the same time slot, they are queued in $Q_{d}^{comp}$, following the rules outlined below:

(1) Priority of offloaded tasks: Tasks that have been offloaded from other devices are given higher priority over tasks that are locally generated at device *d*. This ensures that the device first addresses the tasks that involve inter-device collaboration.
(2) Deadline-oriented queuing: Among the offloaded tasks, those with shorter deadlines are positioned ahead in the queue. This prioritizes tasks that require quicker completion, reflecting the system’s consideration for task urgency.

By adhering to these rules, the system effectively manages task collision and maintains an orderly queue that respects both the source of the tasks and their respective deadlines.

### 3.3. Task Offloading Model

We assume that cellular links and D2D links operate on different frequency bands and adopt orthogonal frequency division multiple access (OFDMA) for access. Therefore, communication between any two devices does not interfere with the communication between other devices [15]. The transmission queue $Q_{d}^{tran},d\in \left[1,D\right]$ also follows the FIFO principle. Similar to the computation queue, let $l_{m}^{tran}\left(t\right)$ denote the slot when $w_{m}\left(t\right)$ is successfully transmitted to the target device or dropped, from when it entered the queue $Q_{d}^{tran}$, so that we obtain the amount of time slots that $w_{m}\left(t\right)$ will wait before transfer in $Q_{d}^{tran}$, as follows:

(3) $$\phi_{m}^{tran}\left(t\right)={\left[\max_{t^{\prime }\in \left\{0,1,\cdots ,t-1\right\}}l_{m}^{tran}\left(t^{\prime }\right)-t+1\right]}^{+}$$

where $l_{m}^{tran}\left(t\right)$ represents the time slot when task $w_{m}\left(t\right)$ leaves the queue $Q_{d}^{tran}$, and we set $l_{m}^{tran}\left(0\right)=0$ for presentation simplicity. The term $\max_{t^{\prime }\in \left\{0,1,\cdots ,t-1\right\}}l_{m}^{tran}\left(t^{\prime }\right)$ determines the time slot when all the tasks placed in the transmission queue before time slot *t* have either been processed or dropped.

In mobile device m∈[1,D], task $w_{m}\left(t\right)$, placed in the transmission queue at the beginning of time slot *t*, needs a conditional part for if task $w_{m}\left(t\right)$ is either completely sent or dropped in time slot $l_{m}^{tran}\left(t\right)$, as follows:

(4) $$\begin{array}{c} l_{m}^{tran}\left(t\right)=\min\left\{t+\phi_{m}^{tran}+\underset{\theta }{\mathrm{argmin}}\left\{s_{m}^{t}\leq \sum_{i=t}^{t+\theta }r_{m}\left(i\right)\Delta t\right\},t+\tau_{m}^{t}-1\right\} \end{array}$$

where $r_{m}\left(t\right)$ represents the transmission rate of MD *m* in *t*. Specifically, the task $w_{m}\left(t\right)$ will start to transmit at the beginning of the time slot $t+\phi_{m}^{tran}\left(t\right)$. The term $\underset{\theta }{\mathrm{argmin}}\left\{s_{m}^{t}\leq \sum_{i=t}^{t+\theta }r_{m}\left(i\right)\Delta t\right\}$ represents the total time slots required to transfer data successfully, where $r_{m}\left(i\right)\Delta t$ represents the transfer data size in slot *i*. Hence, $l_{m}^{tran}\left(t\right)$ decides the time slot when a task will either be sent successfully or dropped.

The transmission rate $r_{m}\left(t\right)$ of MD *m* at *t* can be calculated based on Shannon’s theorem, as follows:

(5) $$r_{m}\left(t\right)=B_{m}\left(t\right)\log_{2}\left(1+\frac{p_{m}h_{m}^{d}\left(t\right)}{N_{0}}\right)$$

where $B_{m}\left(t\right)$ is the bandwidth allocated to MD *m* and $p_{m}$ represents the transmission power of device *m*, which is a constant value. $h_{m}^{d}\left(t\right)$ represents the channel gain between device *m* and target device *d* at time slot *t*, which keeps it fixed within a time slot and follows a Rayleigh distribution that varies over time. $N_{0}$ represents the white noise during transmission. Here, we adopt the average bandwidth allocation scheme, wherein the total bandwidth is allocated equally among each pair of communicating nodes.

Due to the negligible size of task results compared to the original task data, the transmission time for task result return is extremely short. Therefore, similarly to [20], the transmission time for the task result return is ignored.

### 3.4. Task Delay Model

Let $a_{m}\left(t\right)=m$ denote the target device to execute task $w_{m}\left(t\right)$; we have $a_{m}\left(t\right)\in \mathrm{ID}\left(t\right)⋃\left\{m\right\}$.

If $a_{m}\left(t\right)=m$, which means task $w_{m}\left(t\right)$ will be processed locally, based on Equation (2), we have the total delay $L_{m}\left(t\right)=l_{m}^{comp}\left(t\right)-t+1$, which can be written as follows:

(6) $$L_{m}^{loc}\left(t\right)=\min\left\{\phi_{m}^{comp}\left(t\right)+\left\lceil \frac{s_{m}^{t}c_{m}^{t}}{f_{m}\Delta t}\right\rceil ,\tau_{m}^{t}\right\}$$

If $a_{m}\left(t\right)\neq m$, then task $w_{m}\left(t\right)$ will be offloaded to remote device *d* to process. As the transmission delay is $L_{m}^{tran}\left(t\right)=l_{m}^{tran}\left(t\right)-t+1$, the processing delay is $L_{m}^{comp}\left(t\right)=\phi_{m,d}^{comp}\left(t\right)+\left\lceil \frac{s_{m}^{t}c_{m}^{t}}{f_{m}\Delta T}\right\rceil$. Hence, we obtain the following equation for the total delay of the remotely executed task:

(7) $$L_{m}^{rem}\left(t\right)=\min\left\{L_{m}^{tran}\left(t\right)+L_{m}^{comp}\left(t\right),\tau_{m}^{t}\right\}$$

Therefore, the total delay of task $w_{m}\left(t\right)$ can be derived as follows:

(8) $$L\left(m,t\right)=1_{\left(a_{m}\left(t\right)=m\right)}L_{m}^{local}\left(t\right)+1_{\left(a_{m}\left(t\right)\neq m\right)}L_{m}^{rem}\left(t\right)$$

where 1(·) is an indicator function that outputs 1 when · is true and 0 otherwise.

## 4. Problem Formulation

In this paper, we aim to minimize the long-term average task delay under deadline constraints by making task offloading decisions $A\left(t\right)=\{a_{1}\left(t\right),a_{2}\left(t\right),\ldots ,a_{d}\left(t\right)\}$ for each MD in each time slot *t*. The objective function at slot *t* is written as 1D∑d=1DLd,t. Therefore, the task offloading optimization can be formulated as follows:   

$$\begin{array}{cc} \; & \left(P1\right)\;\;\min_{A(t)}\frac{1}{T}\sum_{t=1}^{T}\frac{1}{D}\sum_{d=1}^{D}L\left(d,t\right)\\ s.t. \end{array}$$

(9a) $$\begin{array}{cc} \; & L\left(d,t\right)\leq \tau_{d}^{t},\forall d\in \left[1,D\right] \end{array}$$

(9b) $$\begin{array}{cc} \; & a_{d}\left(t\right)\in \mathrm{ID}\left(t\right)⋃\left\{d\right\},\forall d\in \left[1,D\right] \end{array}$$

In problem P1, constraint (9a) ensures that tasks are completed or dropped upon reaching their deadlines, minimizing average task delay. Constraint (9b) specifies the target device for task execution, including both idle devices and the local device where the task is generated.

Problem P1 can be classified as a dynamic assignment problem, characterized by its complexity in high-dimensional space and being non-deterministic polynomial (NP)-hard. The time complexity of this problem increases exponentially with the cardinality of the sets of available devices |AD(t)| and idle devices |ID(t)| at each time slot *t*. Additionally, the strong coupling of action decisions across multiple time slots exacerbates the computational challenge.

Even if the offline information of the system is known in advance, it is challenging to solve using traditional techniques due to the curse of dimensionality. Hence, we propose a novel algorithm with low complexity that operates online using a multi-agent DRL framework.

## 5. Algorithm Design

Proximal policy optimization (PPO) is a robust policy gradient algorithm within the actor–critic framework, demonstrating exceptional performance in diverse reinforcement learning tasks [36]. Its simplicity and effectiveness have made it a popular choice for numerous RL applications, addressing the stability and sample efficiency concerns of traditional policy gradient methods through careful policy network update clipping and the use of a surrogate objective function.

Beyond the scope of single-agent DRL methods, MAPPO significantly expands the applicability of PPO. It excels in scenarios where multiple agents must interact and make decisions autonomously, demonstrating superior performance in environments that require stability and sample efficiency within discrete action spaces. The robust handling of discrete action spaces and overall resilience in multi-agent systems make MAPPO an ideal choice for the dynamic task offloading problem explored in this study.

This advancement underscores the importance of MAPPO in facilitating collaborative actions among agents, ensuring that each operates optimally within a shared environment. The scalable and efficient solution MAPPO offers is particularly significant for cooperative decision-making in complex settings, addressing challenges that extend beyond the reach of single-agent approaches.

In this section, we present a novel framework for task offloading based on the multi-agent DRL technique. Leveraging the MAPPO technique, our proposed algorithm builds upon the cooperative decision-making abilities of MAPPO to effectively address the dynamic offloading challenges in MEC systems. By formulating the problem as a cooperative Markov game and employing CTDE architecture, our algorithm provides a robust and efficient solution for dynamic offloading in MEC systems, effectively managing the heavy communication burden and offering scalability in decision-making processes.

Firstly, we formulate the problem of minimizing long-term average task delay as a cooperative Markov game.

### 5.1. MDP of P1

#### 5.1.1. State

Our algorithm incorporates both local and global states. The local state includes the system information of each MD and is used to train the actor network for individual decision-making. On the other hand, the global state comprises the entire system information at the current time slot and is utilized to train the critic network.

At the beginning of slot *t*, each device m∈[1,D] observes its state information, which includes task properties such as task size, task complexity, and expiration time, as well as information about the computation queue, transmission queue, and channel state. Let $B_{m}^{comp}\left(t\right)=\max_{t^{\prime }\in 0,1,\cdots ,t-1,m^{\prime }\in \left[1,D\right]}l_{m^{\prime },m}^{comp}\left(t^{\prime }\right)-t+1$ denote the backlog of computation queue at slot *t*, and $B_{m}^{tran}\left(t\right)=\max_{t^{\prime }\in 0,1,\cdots ,t-1}l_{m}^{tran}\left(t^{\prime }\right)-t+1$ denote the backlog of the transmission queue of each MD; we obtain the local state vector as follows:

(10) $$S_{m}\left(t\right)=\left(s_{m}^{t},c_{m}^{t},\tau_{m}^{t},B_{m}^{comp}\left(t\right),B_{m}^{tran}\left(t\right),h_{m}\left(t\right),\vec{id(t)}\right)$$

where $\vec{id(t)}$ is a one-dimension vector and consists of the identifier (ID) of all the idle devices. We assume that each idle MD will broadcast the state of its computation queue at the end of each time slot. For device *m* at time slot *t*, if no task is generated, $s_{m}^{t},c_{m}^{t},and\tau_{m}^{t}$ are all set to zero.

The global state integrates the local state information from each MD and the queue information from the edge server, which are denoted as follows:

(11) $$S\left(t\right)=\left(S_{1}\left(t\right),S_{2}\left(t\right),\ldots ,S_{D}\left(t\right)\right)$$

#### 5.1.2. Action

At the beginning of slot *t*, when MD *m* generates a task $w_{m}\left(t\right)$, the scheduler decides whether to execute the task locally or offload it to an idle device. Therefore, the action vector of each MD can be presented as follows:

(12) $$A\left(t\right)=\left(id_{0},id_{1},id_{2},\ldots ,id_{\mathrm{ID}}\right)\cup m$$

#### 5.1.3. Reward

According to problem P1, the optimization objective is the ratio of the total task delay to the task deadline. Therefore, the reward signal can be directly defined as follows:

(13) $$r\left(t\right)=\frac{\sum_{m\in [1,D]}1_{\left(a_{m}^{t}=m\right)}L_{m}^{local}\left(t\right)+1_{\left(a_{m}^{t}\neq m\right)}L_{m}^{rem}\left(t\right)}{\sum_{m\in [1,D]}1\left(s_{m}^{t}\right)\neq 0)}$$

where the item $\sum_{m\in [1,D]}1\left(s_{m}^{t}\right)\neq 0)$ represents all tasks generated in time slot *t*.

### 5.2. Multi-Agent DRL-Based Algorithm

**Figure 2:**
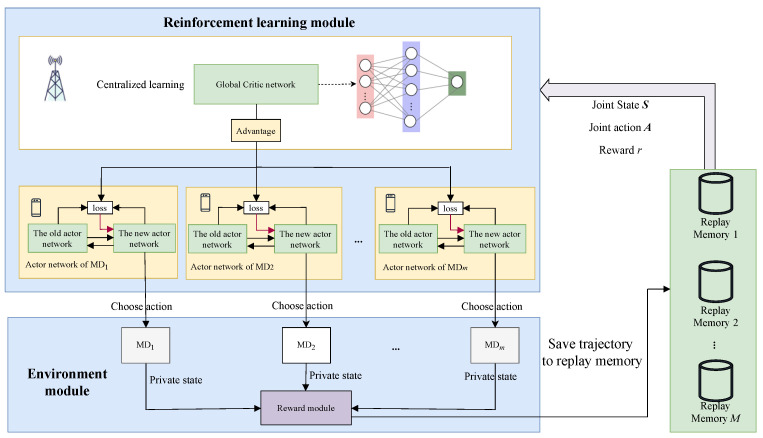
Architecture of multi-agent DRL algorithm.

To reduce the exponential decision space of P1, we propose a multi-agent DRL-based algorithm that leverages a state-of-the-art multi-agent RL technique named MAPPO. MAPPO exhibits significantly higher algorithmic runtime efficiency than and comparable data sample efficiency to off-policy algorithms under limited computing resources, which make it well-suited for a MEC system. Here, we adopt the popular CTDE architecture, which consists of two crucial components: one central critic network and multiple actor networks. The architecture of the multi-agent DRL algorithm is depicted in Figure 2. The process involves two phases, which are described below.

(1) Centralized training
  We utilize a global critic network to obtain accurate evaluations of the global system states to guide the training of actor networks for each MD. Similar to the PPO algorithm, these networks are trained using the following steps:
  (a) Firstly, each agent *m* interacts with the environment by randomly sampling actions based on its observed system state $S_{m}\left(t\right)$ and executing the selected action. Then, agents observe the local state in the next time slot and obtain the reward $r_{m}\left(t+1\right)$. The trajectory data $\left(S_{m}\left(t\right),a_{m}\left(t\right),r_{m}\left(t+1\right),S_{m}\left(t+t\right)\right)$ are stored in the local experience replay pool.
    Afterwards, agent *m* sends its local state $S_{m}\left(t\right)$, the next time step state $S_{m}\left(t+1\right)$, and the local reward $r_{m}\left(t+1\right)$ to the central controller for further processing.
  (b) By aggregating the information from each agent, we acquire the global state S(t), S(t+1), and the global reward r(t). These values serve as inputs for the critic network when computing the state values V(S) and $V(S^{\prime })$. Here, we employ a target network to compute the temporal difference (TD) target and TD error $\delta_{t}=r\left(t\right)+\gamma V\left(S\left(t+1\right)\right)-V(S\left(t\right))$, where γ is the discount factor.
    In the training phase, the system samples a batch of trajectories from the replay memory in the central controller and performs updates on the critic network. The update equation for the critic network can be expressed as follows:
    (14) $$\begin{array}{c} L\left(\phi_{cri}\right)=E_{S(t),A(t)}[r\left(t\right)+\gamma V_{\phi_{cri}}\left(S\left(t+1\right)\right)-V_{\phi_{cri}}{\left(S\left(t\right)\right)]}^{2} \end{array}$$
    where $V_{\phi_{cri}}\left(.\right)$ is the value function of the critic network under parameter $\phi_{cri}$.
    We employ generalized advantage estimation (GAE) to compute the advantage function, as follows:
    (15) $${\hat{A}}_{t}=\sum_{l=0}^{\infty }{\left(\gamma \lambda \right)}^{l}\delta_{t+l}$$
    where γ is used to determine the importance given to future rewards, while λ is a parameter similar to TD(λ), with a trade-off between variance and bias. $\delta_{t+l}^{V}$ refers to the l−step TD error, which is computed as $\delta_{t+l}=r\left(t+l\right)+\gamma V\left(t+l+1\right)-V\left(t+l\right)$.
    The value of GAE will be broadcasted to each agent for the training of the actor network.
  (c) Upon receiving the advantage function ${\hat{A}}_{t}$, each agent conducts batch sampling from its own experience replay pool and calculates the surrogate value by considering the probability distribution of old and new actions, along with the advantage function ${\hat{A}}_{t}$. This surrogate value is used to update the parameters of the actor network. The update equation for the actor network at agent *m* is as follows:
    (16) $$\begin{array}{c} L\left(\theta_{m}^{act}\right)=E_{\pi_{\theta_{m}}}\left[\min\left(\psi_{t}\left(\theta_{m}^{act}\right){\hat{A}}_{t},\mathrm{clip}\left(\psi_{t}\left(\theta_{m}^{act}\right),1-\epsilon ,1+\epsilon \right){\hat{A}}_{t}\right)\right] \end{array}$$
    where $\psi_{t}\left(\theta_{m}^{act}\right)=\frac{\pi_{\theta_{m}}\left(a_{m}\left(t\right)|S_{m}\left(t\right)\right)}{\pi_{\theta_{m}}^{old}\left(a_{d}\left(t\right)|S\left(t\right)\right)}$ represents the probability ratio, ϵ is a hyperparameter in PPO that limits the deviation between the new and old networks, and ${\hat{A}}_{t}$ is used to assess the quality of action A(t) in state S(t).
(2) Decentralized Execution
  After completing centralized training, the critic network becomes unnecessary. The actor network is deployed to each individual MD and utilizes locally observed system states for decision-making. Communication is not required during the decision-making process. Decentralized decision-making is fast and can be performed in real time. The details of our MARL dynamic offloading algorithm are summarized in Algorithm 1.

**Algorithm 1:** Multi-agent DRL-based dynamic offloading algorithm

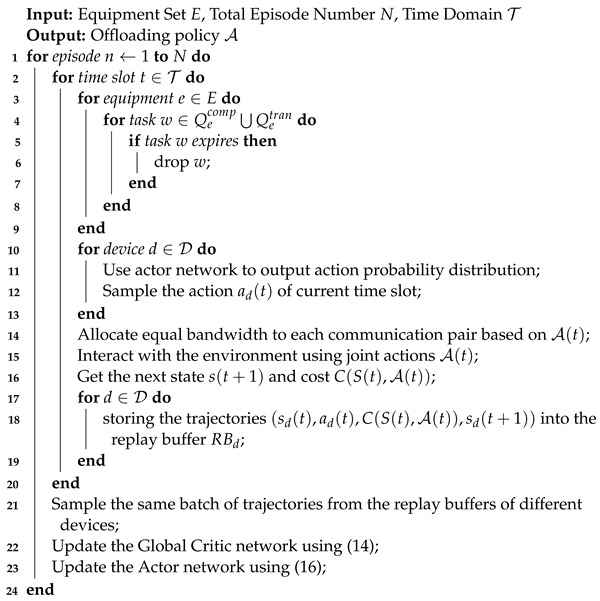



### 5.3. Communication Overhead Analysis

In the multi-agent reinforcement learning model we propose, the CTDE paradigm is adopted, where the communication overhead between each agent and the central controller is primarily concentrated in the training phase. Below, we analyze the system’s communication load during both the training and decentralized execution phases.

During the training phase, the exchange of information among devices is orchestrated as follows:

(1) Idle MDs state information collection: At the end of each time slot, if a MD identifies an empty computation queue, it transmits its identifier to the edge server. The server then designates the MD as idle. After aggregating idle MD status information, the server broadcasts this data to all active MDs. The communication overhead in this phase is minimal, as it only includes the identifiers of idle MDs.
(2) Interaction of active MDs with environment and reporting of system state formation: Upon the receipt of the aggregated information of the idle MD sets, each active MD integrates this data with its local state information to construct a comprehensive system state representation. During time slot *t*, the active MD interacts with the environment, collects current observations denoted as $S_{i}\left(t\right)$, and makes decisions based on its established policy. At the next time step, each agent observes the new state, $S_{i}\left(t+1\right)$ and receives the corresponding reward, $r_{m}\left(t\right)$. These agents then relay $S_{i}\left(t\right)$, $S_{i}\left(t+1\right)$, and $r_{m}\left(t\right)$ back to the central controller located at the edge server.
  The communication overhead in this phase arises from the volume of data transmitted by the agents on each active MD to the edge server. According to the definition of the local system state of the active MDs as mentioned earlier, this communication overhead, after data encoding, can be approximately a few tens of bytes. This is considered a relatively small and acceptable overhead.
(3) Critic network prediction and TD error calculation: The edge server uses the collected data to construct the global system state, predict outcomes, determine the TD target, and compute the TD error. This TD error is then broadcast to all agents on active MDs, thereby aiding in the update of the parameters within the value network. The communication overhead at this juncture is solely due to the broadcast of the TD error, which involves a minimal amount of data transmission, exerting virtually no impact on network communication.
(4) Policy network update: With the TD error received, each active MD’s agent refines its policy network, adapting to the new information and improving its decision-making process for future interactions. No communication overhead is incurred at this stage.
  During the decentralized execution phase, each agent makes decisions solely based on its current system state, eliminating the need for the central value network. Consequently, the communication overhead at this stage is primarily attributed to the acquisition of information regarding idle MDs. According to the aforementioned analysis, the communication overhead generated from obtaining the status of idle MDs is minimal and does not adversely affect system performance. This reduction is particularly beneficial in maintaining the efficiency and responsiveness of the system, especially in scenarios where resource conservation is critical.

## 6. Simulation Results

### 6.1. System Parameter Settings

To verify our algorithm, we conducted extensive simulations, demonstrating its convergence and performance superiority compared to baseline algorithms. Table 2 lists the basic system parameters.

The centralized critic networks and the actor network in each MD utilize a three-layer network structure, with 64 nodes in the intermediate layer. During the neural network training process, a batch size of 64 is employed, the learning rate is set to 1 × 10^3^, and the number of MDs is fixed at 20. The task generation probability gradually increases from 0.1 to 0.5. Network parameters are updated using the Adam optimizer. To ensure high randomness and unpredictability in the experimental data generation, the relationship between random numbers and the task generation probability determines whether a task is generated. The channel gains between devices follow the Rayleigh distribution.

### 6.2. Algorithm Convergence Performance

**Figure 3:**
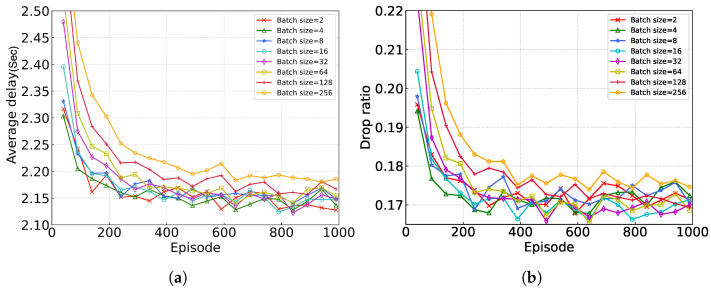
Performance evaluation under different batch sizes. (**a**) average delay; (**b**) ratio of dropped tasks.

In this section, we mainly study the convergence performance of the proposed algorithm under different values of different hyper-parameters. We consider 1000 episodes and each episode has 100 time frames. The experimental results are shown in Figure 3, where the *X*-axis represents the episodes and the *Y*-axis represents the average completion delay of the task (the average time required by the task from creation to completion).

Figure 3 shows the convergence of the proposed algorithm under different batch sizes, which refer to the number of training examples utilized in one iteration. Generally, as the batch size increases, the gradient estimation during each training step becomes more accurate, thereby reducing the variance of parameter updates. However, larger batch sizes may make the model more prone to getting stuck in local minima and lose some ability to escape from them. On the other hand, smaller batch sizes can provide more randomness, helping the model to jump out of local minima. As we can see from Figure 3, changing the parameter of the batch size has only about a 1% impact on performance. Thus, we choose an appropriate batch size (e.g., 64) to increase the convergence speed without reducing the performance significantly.

**Figure 4:**
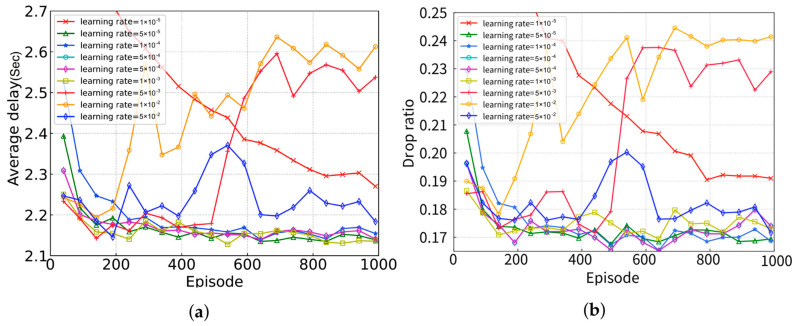
Performance evaluation under different learning rates. (**a**) average delay; (**b**) ratio of dropped tasks.

Figure 4 shows the convergence of the proposed algorithm under different learning rates, which is a tuning parameter in an optimization algorithm that determines the step size at each iteration while moving toward the minimum of a loss function. In Figure 4, when the learning rate is too small (e.g., 1 × 10^5^), it will lead to a relatively slow convergence and, therefore, requires more computational resources. However, if the learning rate is too large (e.g., 1 × 10^2^, 5 × 10^2^), the neural network cannot converge to a good performance because of the great vibration of loss function.

**Figure 5:**
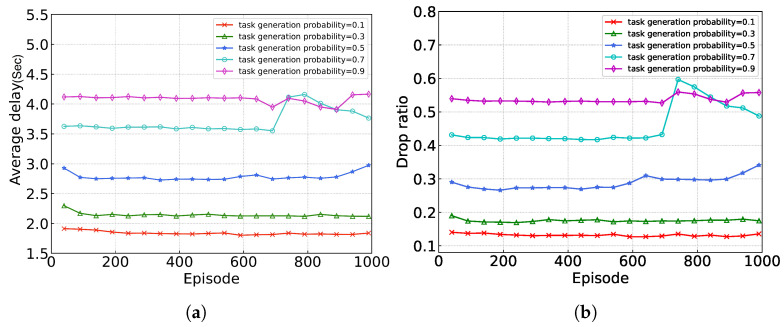
Performance evaluation under different task generation probabilities. (**a**) average delay; (**b**) ratio of dropped tasks.

Figure 5 shows the convergence of the proposed algorithm under different task generation probabilities, where the task generation probability is the probability of each device producing a task in each time frame. As shown in Figure 5, there is an obvious correlation between task generation probability and algorithm performance: the larger the task generation probability, the larger the average delay. This is because, with limited computing and communication resources, the higher the probability of task generation, the fewer resources are allocated on average, and the higher the delay of task completion.

**Figure 6:**
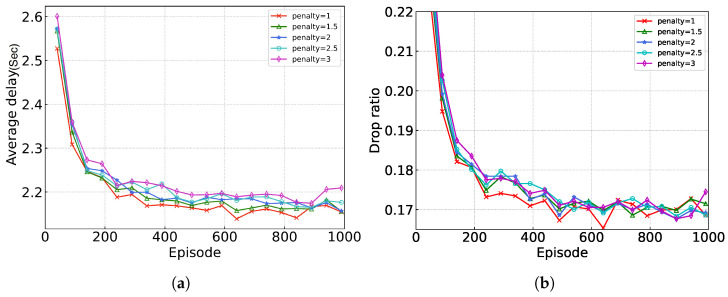
Performance evaluation under different penalties. (**a**) average delay; (**b**) ratio of dropped tasks.

Figure 6 illustrates the performance of the proposed algorithm under different drop penalty values, where the penalty values are represented as follows:

(17) $$C\left(S\left(t\right),A\left(t\right)\right)=\left\{\begin{array}{cc} Penalty\;Value, & \mathrm{if}\;\mathrm{task}\;\mathrm{is}\;\mathrm{dropped}\\ \frac{1_{\left(a_{m}\left(t\right)=m\right)}L_{m}^{local}\left(t\right)+1_{\left(a_{m}\left(t\right)\neq m\right)}L_{m}^{rem}\left(t\right)}{\tau_{m}^{t}}, & \mathrm{if}\;\mathrm{task}\;\mathrm{is}\;\mathrm{not}\;\mathrm{dropped} \end{array}\right.$$

Generally, as the penalty value increases, the model should become prone to dropout, which thus results in a decrease in the drop ratio. Interestingly, the experimental results show the opposite behavior. This may be due to the change from a continuous range of penalty values with an upper limit of one to discrete values. This change may make it difficult for the critic network to converge, resulting in the observed results.

### 6.3. Performance Comparison Evaluation

To valuate the performance of our algorithm, we consider the following benchmark algorithms:

(1) IPPO: each agent employs an independent PPO algorithm to select actions based on global state information;
(2) A2C: the A2C algorithm is used to sample from ${\left(1+N\right)}^{N}$ types of actions;
(3) TD3: the multidimensional discrete action space is transformed into multiple continuous actions, and the selection of an action is based on the shortest Euclidean distance [2];
(4) Heuristic: if a task can be completed within two time slots, it is computed locally; otherwise, it is offloaded to the edge server;
(5) All_local: all tasks are executed locally;
(6) All_edge: all tasks are offloaded to the edge server;
(7) Greedy: the action with the shortest expected time, considering the current queue situation and channel status, is selected;
(8) Random: Actions for each task are randomly selected. Here, we use two performance metrics: the average delay and the drop ratio(ratio of the number of timeout tasks to the total number of tasks.

Although MADDPG [31] is a representative and high-performing algorithm in the field of multi-agent reinforcement learning, it was not selected as a benchmark in this study for specific reasons. The MADDPG algorithm is primarily tailored to optimize continuous action spaces, which does not align perfectly with the discrete action space of the problem addressed in our study.

**Figure 7:**
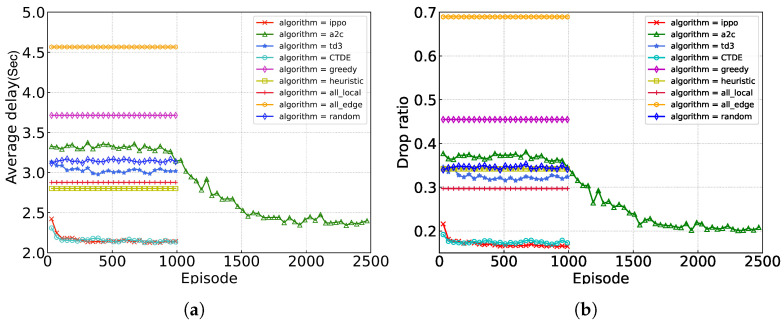
Performance of different algorithms. (**a**) average delay; (**b**) ratio of dropped tasks.

Figure 7 illustrates the convergence of our proposed algorithm and baseline algorithms. It can be observed that our algorithm presents a similar performance to the IPPO algorithm, in terms of the delay metric. However, our algorithm only relies on local state information for decision-making, significantly reducing communication overhead. In comparison to the sub-optimal A2C algorithm, our approach achieves an 11.0% reduction in the delay metric and a 17.0% reduction in the drop ratio metric.

**Figure 8:**
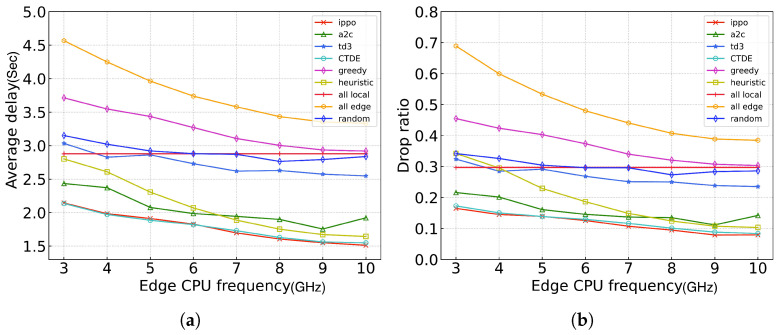
Performance of different algorithms across different edge CPU frequencies. (**a**) average delay; (**b**) ratio of dropped tasks.

Figure 8 illustrates the comparison of the average delay and drop ratio between our proposed algorithm and the baseline algorithms under different edge server frequencies. As the computing power of the edge server increases, the overall system computing resources increase, resulting in a decreased average delay and drop ratio. It is important to note that the all_local algorithm, which does not rely on edge servers, maintains a stable performance curve.

**Figure 9:**
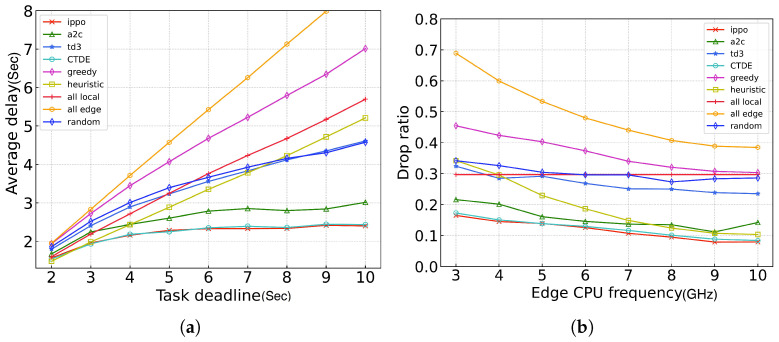
Performance of different algorithms across different task deadlines. (**a**) average delay; (**b**) ratio of dropped tasks.

Figure 9 shows the comparison of the average delay and drop ratio between our proposed algorithm and the baseline algorithms under different task deadlines. From Figure 9, it is evident that several benchmark algorithms, such as all_local, all_edge, and greedy, face a limitation imposed by the deadline, causing the average delay to approach the upper limit, i.e., the deadline itself. Consequently, as the deadline increases, the average delay also grows, while the drop ratio decreases due to the slack in the deadline. In contrast, the CTDE, IPPO, and A2C algorithms exhibit superior performances. When the deadline is less than five, their curves resemble those of other algorithms, albeit relatively flat. However, as the deadline continues to increase, these algorithms reach a stable curve, suggesting that the tasks have been fully processed.

## 7. Conclusions

In this paper, we investigate the dynamic task offloading problem in D2D–MEC systems for delay-sensitive tasks under delay constraints. We propose a dynamic partitioning approach for a D2D MD set based on a queuing-based system. We formulate the minimization of a long-term average task delay with deadline constraints as a cooperative Markov game and propose a multi-agent DRL-based algorithm. Our proposed algorithm is implemented in a CTDE-based manner, which enables each MD to make offloading decisions solely based on its local system state. Extensive simulations show the efficiency of our algorithm. In future work, we will consider bandwidth resource allocation strategy in the D2D–MEC network.

## Figures and Tables

**Table 1:** Key Notations.

Symbol	Definition
D	The set of mobile devices
T	The whole time slots
Δt	The duration of time slot *t*
$w_{m}\left(t\right)$	The computation task generated on mobile device *m* at time slot *t*
$s_{m}^{t}$	The size of task $w_{m}\left(t\right)$
$c_{m}^{t}$	The computation complexity of task $w_{m}\left(t\right)$
$\tau_{m}^{t}$	The deadline for task $w_{m}\left(t\right)$
A(t)	The task offloading decision for all MDs at time slot *t*
$a_{m}^{t}\left(t\right)$	The offloading decision of the *m*th active device at time slot *t*
$Q_{d}^{comp}$	The computing queue of mobile device *d*
$Q_{d}^{tran}$	The transmission queue of mobile device *d*
$l_{m,d}^{comp}\left(t\right)$	The time slot when task $w_{m}\left(t\right)$ is fully processed at mobile device *d*
$l_{m}^{tran}\left(t\right)$	The time slot when task w(m,t) transmission is completed at device *m*
$\phi_{m,d}^{comp}\left(t\right)$	The waiting time slots of task $w_{m}\left(t\right)$ in computation queue at mobile device *d*
$\phi_{m}^{tran}\left(t\right)$	The waiting time slots of task $w_{m}\left(t\right)$ in transmission queue at mobile device *m*
$r_{m}\left(t\right)$	The transmission rate of mobile device *m* at time slot *t*
$p_{m}$	The transmission power at device *m*
$h_{m}^{d}\left(t\right)$	The channel gain between active device *m* and idle device *d* at slot *t*
$N_{0}$	The white noise
$B_{m}\left(t\right)$	The bandwidth of device *m* at time slot *t*
L(m,t)	The total duration of task w(m,t) from generation to execution completion
$S_{m}\left(t\right)$	Local observation information of device *m* on time slot *t*
S(t)	Global state information on time slot *t*
r(t)	Reward in time slot *t*

**Table 2:** Simulation system parameters.

Parameters	Values
Mobile device number *D*	20
The CPU frequency of mobile device $f_{d}$	2 GHz
The CPU frequency of edge server $f_{0}$	3 GHz
Minimum task size $s_{min}$	3 Mbits
Maximum task size $s_{max}$	10 Mbits
Minimum task complexity $c_{min}$	0.5 gigacycles per Mbits
Maximum task complexity $c_{max}$	2 gigacycles per Mbits
Minimum task generation probability	0.1
Maximum task generation probability	0.5
Total bandwidth *B*	3 MHz
Device transmission power $p_{d}$	3 W
White noise $N_{0}$	−14 dbm\Hz

## Data Availability

Available on request.
